# Viral Myositis in an Eight-Year-Old

**DOI:** 10.7759/cureus.56887

**Published:** 2024-03-25

**Authors:** Micah Pippin, William Stansbury, Praveen Budde

**Affiliations:** 1 Family Medicine, Louisiana State University Health Sciences Center, Alexandria, USA

**Keywords:** stiff-legged gait, parainfluenza, viral infection, benign acute childhood myositis, viral myositis

## Abstract

Benign acute childhood myositis (BACM) is a pediatric syndrome characterized by mild self-limiting sudden onset of muscle pain during or following recovery from a viral illness. The case discussed in this report is of an eight-year-old female diagnosed with the common cold after presenting to her primary care physician. Five days later, the patient presented to the emergency department with lower extremity pain. The patient was sent home with supportive care and mild analgesics. Twelve hours later, the patient was seen again in the emergency department with severe bilateral lower extremities pain and difficulty walking. BACM most commonly affects school-age children and is usually caused by influenza A and B. The main goal of this case report is to help primary care and emergency medicine physicians diagnose benign acute childhood myositis as early as possible and treat the condition appropriately.

## Introduction

This case report discusses the clinical scenario, multifactorial diagnostic workup, treatment plan, complications, and alternative diagnoses for benign acute childhood myositis. Benign acute childhood myositis (BACM) is characterized by mild to sudden onset of muscle pain during or following recovery from a viral illness [1]. BACM most commonly involves school-aged children and presents with an acute onset of reluctance to walk due to severe lower leg pain, usually lasting three days. Common viral prodromal symptoms such as rhinorrhea, fever, sore throat, and malaise are to be expected [2]. BACM is postulated to be caused by viral invasion of the myocytes and inability to replicate, causing muscular necrosis [3]. BACM is usually self-limiting, requiring minimal workup and supportive therapy only [2].

## Case presentation

An eight-year-old female presented to the clinic with her mother, reporting flu-like symptoms, cough, runny nose, sore throat, and low-grade fever. The patient's mother reported that the symptoms had been present for two days. The child's vital signs were recorded as a blood pressure of 105/69 mm/Hg, heart rate of 116 beats per minute, respirations of 18 breaths per minute, SpO2- 95% on room air pulse oximetry, and a temperature of 100.2^o^F (37.9^o^C). The patient had a history of attention deficit disorder, which had been managed with lisdexamfetamine 20 mg. On physical examination, she had non-purulent mucoid nasal discharge and erythema of the pharynx. The patient was tested for influenza and Streptococcus pyogenes. The rapid influenza and rapid streptococcus diagnostic tests were negative. The patient was diagnosed with a common cold and sent home with supportive care and over-the-counter analgesia with acetaminophen.

Five days after the initial presentation, the patient was seen in the hospital's emergency department with severe lower extremity pain and difficulty ambulating. The mother reported that the patient's initial respiratory symptoms were improving but not entirely resolved. On day four of recovery, the patient started complaining of calf pain bilaterally, rating it at a five on a scale from one to ten. On day five of recovery, the patient started to have worsening difficulty walking. Vital signs were a blood pressure of 109/72 mm/Hg, a heart rate of 120 beats per minute, respirations of 17 breaths per minute, SpO2- 96% on room air pulse oximetry, and a temperature of 99.8^o^F (37.7^o^C). The patient was discharged home from the emergency room with a continuation of supportive care and over-the-counter pain treatment.

Twelve hours after being seen in the emergency department, the patient returned to the hospital again with increased severity of symptoms. The mother then reported that the patient could hardly walk independently and that the pain had increased to nine on a scale of one to ten. On physical examination, there was tenderness to bilateral calves. The patient was reluctant to walk initially but demonstrated a stiff-legged gait after encouragement (Video 1).

**Video 1:** Stiff-legged gait associated with benign acute childhood myositis

An elevated creatinine phosphokinase (CPK) level of 5,518 mcg/L (normal 55-200 mcg/L) and a decreased white blood cell count (WBC) of 3,000 WBC/microliter (normal 4,500-11,000 WBC/microliter) were seen on laboratory findings (Table 1).

**Table 1:** Complete blood count (CBC) and creatinine phosphokinase (CPK)

Laboratory Findings		
Laboratory Values	Patient Values	Reference Ranges
Creatinine Phosphokinase (CPK)	5,518 mcg/L	55-200 mcg/L
White Blood Cells (WBC)	3,000 WBC/mL	4,500-11,000 WBC/mL
Red Blood Cells (RBC)	4.62 million cells/mcL	4.1-5.1 million cells/mcL
Hemoglobin	13.0 gm/dL	12.3-15.3 gm/dL
Hematocrit	41.6%	35.9-44.6%
Mean Corpuscular Volume (MCV)	90 femtoliters (fL)	80-96 femtoliters (fL)
Red Cell Distribution Width (RDW)	12.0%	12-15%
Platelet Count	216,000/mcL	150,000-371,000/mcL
Mean Platelet Volume (MPV)	9.6 femtoliters (fL)	7.4-10.4 femtoliters (fL)

A viral panel was performed and showed a positive result for parainfluenza type 1 (Table 2).

**Table 2:** Viral panel positive for parainfluenza virus 1 SARS-CoV-2: Severe acute respiratory syndrome coronavirus 2.

Viral Panel	
Virus	Results
Monospot	Negative
Influenza A	Negative
Influenza B	Negative
Adenovirus	Negative
Respiratory Syncytial Virus (RSV)	Negative
Coronavirus HKU1	Negative
Coronavirus NL63	Negative
Coronavirus OC43	Negative
Metapneumovirus	Negative
Parainfluenza 1	Positive
Parainfluenza 2	Negative
Parainfluenza 3	Negative
Parainfluenza 4	Negative
Rhino/Enterovirus	Negative
SARS-CoV-2	Negative

Dipstick urinalysis was positive for blood, but no red blood cells were present on urine microscopy (Table 3). 

**Table 3:** Dipstick urinalysis positive for hemoglobin

Urinalysis	
Laboratory Values	Patient Values
Color	Straw
Clarity	Clear
Specific Gravity	1.006 (normal 1.010-1.020)
Glucose	Negative
Protein	Negative
Bilirubin	Negative
Urobilinogen	Negative
pH	7.0 (normal 6.0-7.5)
Hemoglobin	Positive
Ketones	Negative
Nitrites	Negative
Leukocyte Esterase	Negative

A computed tomography (CT) scan of the abdomen and pelvis was also obtained, suggesting mesenteric adenitis with clusters of mildly enlarged lymph nodes in the right lower quarter mesentery, most likely caused by a viral syndrome. The patient was admitted to the pediatric intensive care unit (PICU) for observation for possible rhabdomyolysis and was diagnosed with BACM. In the PICU, the patient was treated with aggressive intravenous fluid replacement to prevent kidney damage, and her pain was managed with ibuprofen 200 mg twice a day. One day later, the CPK trended down to 3,777 mcg/L, lower leg pain improved to three out of ten, and her gait improved significantly. After three days in the hospital, the patient had almost fully recovered with minimal pain, a normal gait, and a CPK of 1,307 mcg/L. The patient was then discharged with instructions to follow up with her primary physician in one week.

## Discussion

Benign acute childhood myositis is characterized by the sudden onset of muscle pain, primarily calf pain, during or following recovery from a viral illness [1]. BACM was first reported as "myalgia cruris epidemica” in a study conducted by Lundberg in 1957 [4-5]. He observed school-aged children with severe calf myalgia following an upper respiratory infection [4-5]. BACM most commonly involves school-aged children, and boys are more commonly affected [1]. Sudden onset with reluctance to walk due to severe lower leg pain usually lasts for three days after a resolving viral illness, which was within the window of our patient. Common viral prodromal symptoms such as rhinorrhea, fever, sore throat, and malaise are expected [2]. BACM is thought to be caused by viral invasion of the myocytes and the inability to replicate, causing muscular necrosis [3]. BACM is usually self-limiting and needs minimal workup (Table 4) [2]. 

**Table 4:** Viruses associated with benign acute childhood myositis [1], BACM: Benign acute childhood myositis.

BACM Associated Viruses
Influenza A and B
Parainfluenza
Coxsackie
Herpes Simplex
Epstein-Barr
Adenovirus

The most common virus associated with BACM is influenza A and B, which we ruled out with a viral panel in our patient. Influenza virus infections commonly occur in the winter and in the northern hemisphere. Common clinical signs and symptoms include headache, fever, cough, and rhinorrhea. Myalgia is also a common influenza symptom but distinctly differs from BACM muscle pain, which has a later onset, focuses on the lower extremities, and is more severe. Influenza cases usually manifest as either upper respiratory infections or pneumonia. Influenza B is more likely to become BACM because of the complex protein that it employs for viral entry [3,6].

A diagnosis of BACM is made through clinical presentation, history, and laboratory findings. A primary complaint of pain in bilateral calves should bring BACM to the clinician’s mind; however, other more life-threatening diseases should also be considered. The differential diagnosis for BACM includes Guillain-Barre syndrome, rhabdomyolysis, juvenile idiopathic arthritis, malignancy, dermatomyositis, polymyositis, muscular dystrophy, and trauma (Table 5) [1].

**Table 5:** Differential diagnosis for benign acute childhood myositis [1-2,5]

Differential Diagnosis for BACM
Guillain Barre
Rhabdomyolysis
Juvenile idiopathic arthritis
Malignancy
Autoimmune myositis
Muscular dystrophy
Trauma and fracture

BACM presents as a sudden onset of reluctance to walk with severe lower leg pain, most commonly in the gastrocnemius and soleus complex, usually lasting for three days. Children of school age, most commonly boys, are most likely to acquire BACM; however, our patient was female. Children are less likely to have tenderness to anteromedial thighs and rarely pain to the upper extremities. Pain is most likely to worsen with rest. A recent viral illness history is critical for the diagnosis and was present in our patient, who tested positive for parainfluenza virus [1,5-6]. An elevated CPK level is the most common laboratory finding in BACM. Ninety-five percent of patients demonstrate an elevated CPK, which can rise to 20 times the normal value. In our patient, the CPK level rose to 5,518 mcg/L. Although there are no clear guidelines for investigating BACM, in one study by Agyeman et al., it is suggested to only use CPK levels in patients who have experienced muscle pain for multiple days or if it has worsened. In the same study, investigators expressed the importance of ruling out rhabdomyolysis with urine and renal function studies [1,5]. CPK and urinalysis were both analyzed in our patient because of the high suspicion of rhabdomyolysis.

BACM is self-limiting; patients are expected to recover fully within three days. Most often, only supportive care with analgesics and follow-up appointments are required, and hospitalization is unnecessary [2]. Antivirals can help decrease the duration of the accusing virus but are most likely ineffective due to the late onset of BACM [1]. Our patient was hospitalized for three days, but because of the late diagnosis of BACM, antivirals were not utilized.

Rhabdomyolysis is a rare complication of BACM and is most frequently seen in patients with comorbidities such as renal failure. It occurs more commonly in females, which is the gender of our patient [4]. The pathogenesis of rhabdomyolysis caused by a viral syndrome is still undetermined, but the most common hypotheses suggest a cytokine storm with direct muscle breakdown, viral toxins, and direct viral invasion [7]. One characteristic finding of rhabdomyolysis is the presence of red blood cells on dipstick urinalysis but none seen on urine microscopy [7]. This finding was present in our patient; however, she suffered no complications of rhabdomyolysis, such as organ dysfunction or significant electrolyte disturbances [7]. For patients with rhabdomyolysis, hospitalization is required for monitoring, specifically for renal failure and electrolyte abnormalities [2].

BACM can also precipitate the development of compartment syndrome in extreme cases [8]. Our patient did not demonstrate a constellation of symptoms typical of compartment syndrome, such as paresthesias, pulselessness, pallor, or paralysis [8].

## Conclusions

Benign acute childhood myositis is a commonly overlooked diagnosis in school-aged children with a viral syndrome. BACM is most commonly self-limiting but, under the right circumstances, can result in significant complications. Primary care physicians suspicious of BACM should be cautious, perform a comprehensive examination, and schedule regular follow-ups because of the disease sequelae. The pathogenesis of muscle invasion by the virus is undetermined and would be an excellent research opportunity. Limitations of this study include those inherent to case report design and single patient populations, including the potential for selection bias.
